# Patient Safety in Complementary Medicine through the Application of Clinical Risk Management in the Public Health System

**DOI:** 10.3390/medicines4040093

**Published:** 2017-12-16

**Authors:** Elio G. Rossi, Tommaso Bellandi, Marco Picchi, Sonia Baccetti, Maria Valeria Monechi, Catia Vuono, Federica Sabatini, Antonella Traversi, Mariella Di Stefano, Fabio Firenzuoli, Sara Albolino, Riccardo Tartaglia

**Affiliations:** 1 Tuscan Regional Centre for Integrative Medicine, Region of Tuscany, 50100 Florence, Italy; marpicchi@tin.it (M.P.); sonia.baccetti@uslcentro.toscana.it (S.B.); valeriamonechi@gmail.com (M.V.M.); catia.vuono@uslcentro.toscana.it (C.V.); federica.sabatini@uslcentro.toscana.it (F.S.); antonella.traversi@uslcentro.toscana.it (A.T.); m.distefano@mednat.it (M.D.S.); fabio.firenzuoli@unifi.it (F.F.); 2 Regional Centre for Clinical Risk Management and Patient Safety, 50100 Florence, Italy;bellandit@aou-careggi.toscana.it (T.B.); sara.albolino@gmail.com (S.A.); riccardo.tartaglia@aou-careggi.toscana.it (R.T.)

**Keywords:** complementary and integrative medicine, patient safety, clinical risk management, adverse events, significant event audit (SEA), failure modes and effects analysis (FMEA)

## Abstract

**Aim:** To develop a systematic approach to detect and prevent clinical risks in complementary medicine (CM) and increase patient safety through the analysis of activities in homeopathy and acupuncture centres in the Tuscan region using a significant event audit (SEA) and failure modes and effects analysis (FMEA). **Methods:** SEA is the selected tool for studying adverse events (AE) and detecting the best solutions to prevent future incidents in our Regional Healthcare Service (RHS). This requires the active participation of all the actors and external experts to validate the analysis. FMEA is a proactive risk assessment tool involving the selection of the clinical process, the input of a multidisciplinary group of experts, description of the process, identification of the failure modes (FMs) for each step, estimates of the frequency, severity, and detectability of FMs, calculation of the risk priority number (RPN), and prioritized improvement actions to prevent FMs. **Results:** In homeopathy, the greatest risk depends on the decision to switch from allopathic to homeopathic therapy. In acupuncture, major problems can arise, mainly from delayed treatment and from the modalities of needle insertion. **Conclusions:** The combination of SEA and FMEA can reveal potential risks for patients and suggest actions for safer and more reliable services in CM.

## 1. Introduction

The recent case of an Italian child who died of encephalitis caused by otitis treated exclusively with homeopathic remedies has revived the issue of patient safety with respect to complementary medicine (CM), and in particular, homeopathy [1].

The assessment and management of clinical risk and the prevention of adverse events (AE) are the starting points to ensure an adequate level of patient safety and to foster the professional development of healthcare professionals involved in this context.

In a recent article, Makary et al. (2016) analysed the data from four different studies on the causes of death in hospitalized patients and reported a death rate from adverse events of 0.71. This percentage, calculated with respect to the total number of hospital admissions in the United States in 2013 (35,416,020), with an estimate of 251,454 medical error-related deaths, makes medical error the third leading cause of death after cardiovascular disease and cancer. The authors highlighted the difficulties in finding data on this issue, since the causes of death are coded according to the International Classification of Diseases, ICD 10 system, which only reports on death caused by medical errors (e.g., side effects linked to the use of anticoagulants) and deaths caused by overdoses. The system does not specifically include adverse events [2].

Analysis of the incidents may be subdivided into two major areas: one involves analysing individual factors, and the other studies the systemic features of the healthcare organizations that may contribute to adverse outcomes. Because healthcare organizations involve a large number of variables, more serious incidents with numerous individuals and factors contributing to the onset of the event may occur over longer periods of time. Under these circumstances, organizational analysis with a systemic perspective is highly effective for the interpretation of incidents [3,4].

The primary objective of risk management is to promote a culture of safety. This is not easy to achieve, as the medical culture is still based on cognitive resources and technical skills, whereas it is necessary to develop processes for interpersonal and interdisciplinary collaboration in order to identify protective barriers able to prevent or to correct cognitive or procedural errors that can lead to adverse events.

In complementary medicine, the need to foster strategies of safety and prevention is even stronger for several reasons. Firstly, there is a large number of individuals using these medicines [5]. Secondly, there is a widespread opinion that CM can be inefficient or of little effect, but without adverse effects. Finally, as in the case of the Regional Healthcare System (RHS) of Tuscany in Italy, due to the integration of these treatments in the public health system, constant monitoring of clinical practice is required.

### 1.1. Adverse Effects in CM

According to the literature, the highest risks of adverse events in CM are generally related to medicinal plants and herbs and derive mainly from uncontrolled self-medication: the use of unsafe products containing numerous herbs that are often inadequately prepared or used at an incorrect dosage and/or at times are taken in the presence of specific contraindications. Another danger derives from the simultaneous intake of herbs and drugs: indeed, all medicinal plants and herbs with specific biological activity may interact with drugs, and enhance or reduce intended effects owing to their action on P-glycoprotein and on the microsomal systems of cytochrome P450. Typical examples are *Echinacea angustifolia* (DC) Heller (narrow-leaved coneflower), and grapefruit juice, which increase the bioavailability and toxicity of many drugs (calcium channel blockers, statins, and psychoactive drugs). Another example is *Hypericum perforatum* L. (St. John’s wort), which has enzyme-inducer activity and can interfere with many treatments by reducing the blood levels of many drugs (cyclosporine, digitalis, theophylline, anti-retroviral medications, and oral anticoagulants) [6].

There is a low incidence of acupuncture-related effects compared to other therapies in addition to the total absence of side effects in the diagnostic phase. A systematic review of the articles published over the last 20 years [7] shows that the rate of serious adverse effects is very low (0.020–0.1%) and is mainly related to the insertion of needles by personnel who are not qualified physicians, therefore lacking professional expertise and manual skills.

A similar conclusion was reached by the US National Institute of Health (NIH) during the Consensus Conference on Acupuncture in 1998. Xu et al. (2013) published a review of 117 studies (308 cases) on the adverse effects of acupuncture, moxibustion, and cupping in 25 countries between 2000 and 2011. For acupuncture, the highest number of cases included infections (239). In addition, 13 cases of pneumothorax, 9 lesions of the central nervous system, 4 lesions of the peripheral nerves, and 5 lesions of the heart were also identified. The conclusion was that, although serious adverse events are rare, the practice of acupuncture is not without risks, and guidelines are required in order to reduce adverse events [8].

Homeopathy, similarly to other therapies that use diluted drugs (anthroposophic medicine and homotoxicology), is considered extremely safe. Homeopathic remedies are not toxic and have minimal or nonexistent adverse effects; furthermore, because minimum doses are used, they could also be suitable for pregnant women, newborns, and children.

A survey containing data from 1970 to 1995 [9] reported a higher incidence of adverse effects with homeopathic medicines as compared to placebo. However, these effects were mild and transient, leading the authors to conclude that highly diluted homeopathic drugs prescribed by medical doctors specialized in homeopathy are safe.

A study on the patients of the Clinic of Homeopathy in Lucca, Italy [10], investigated the frequency of adverse events during homeopathic follow-up visits of 335 patients from 1 June 2003 to 30 June 2004. There were nine cases of side effects identified, amounting to 2.68% of all cases (including a case of lactose intolerance). This percentage was in accordance with other information available in the literature [11,12].

The adverse effects related to anthroposophic medicine prescriptions have been analysed in depth.

In 265 studies examined by Kienle et al. (2011) [13], no serious undesirable effects were reported, and in any case, they were rare and generally modest. A study of 715 patients with acute infections of the respiratory tract or of the ear treated with anthroposophic medicine [14], and the Anthroposophic Medicine Outcomes Study (AMOS) of 11,487 patients with the same types of infections treated with 949 different products, reported a very low incidence of adverse reactions (only 0.3% with severe intensity) [15].

### 1.2. CM in the Tuscan Regional Healthcare System

The Tuscany Regional Government provides acupuncture, herbal medicine, and homeopathy to the public within the Regional Healthcare Service (RHS). According to a survey conducted in 2015, there are 91 public clinics which provide complementary medicine: 29 provide acupuncture and Traditional Chinese Medicine (TCM), 18 provide homeopathy, 10 provide herbal medicine, and 8 centres provide other types of therapies. In addition, there are 26 clinical CM activities practiced in public hospitals within the so-called *intramoenia* scheme [16].

In 2005–2007 the Regional Health Plan of Tuscany integrated CM into the RHS, and acupuncture, herbal medicine, homeopathy, and manual medicine were officially recognized in the regional Essential Levels of Assistance (LEA) [17].

Resolution 655 (20 June 2005) included complementary medicines in the official regional price lists (*nomenclatore tariffario*), making it possible for Tuscan citizens to receive specialist treatment in acupuncture, homeopathy, herbal medicine, and manual medicine with a €2400 co-payment fee [18].

### 1.3. Drug Surveillance in the Region of Tuscany

In order to increase the number of health professionals involved in reporting suspected adverse drug reactions (ADR), the Region of Tuscany uses methods that have been proven effective in other European countries by delivering, for example, feedback to the family doctor (GP). A law passed in 2003 also states that regions should have a system of pharmacovigilance (regional pharmacovigilance centres, CRFVs) [19]. In 2004 the Region of Tuscany set up a Tuscan Regional Pharmacovigilance Centre collaborating with the local health units and university hospital authorities [20]. A system of “phytovigilance” available at the centre allows for collection of useful information and notifications on the adverse effects of complementary medicines and the possible interactions of medicinal plants with commonly used drugs [21].

After the implementation of this system, Tuscany achieved excellent quality levels in this field, and was the second-best performing Italian region in 2013, with more than 1600 notifications of ADR reported per million inhabitants. This result is far beyond the gold standard of 300 notifications indicated by the World Health Organization (WHO) [22].

With respect to CMs, with the aim of developing a safety culture, the specific four-day training course “Patient safety and risk management in CMs” organized in 2009 was attended by all the medical doctors and health professionals of CM public centres of the RHS: the results of the course constitute the core content of a book published in 2010 [23].

## 2. Aims

The aim of this study is to set the priority for risk management in acupuncture and homeopathy, which are the CMs most frequently integrated into the RHS of Tuscany. A combination of reactive and proactive methods to prevent AEs in CM was used: the significant event audit (SEA) and failure modes and effects analysis (FMEA).

## 3. Materials and Methods

The development of systems or theories for the prevention of clinical risk in CMs is influenced by a set of elements that can make it very difficult for healthcare providers to identify efficient and easily manageable procedures. Firstly, it is important to achieve a shared terminology for wide-ranging debate on the subject, starting with the definition of the terms provided by the WHO (Table 1).

### 3.1. Significant Event Audit (SEA)

SEA is the selected tool for studying adverse events (AE) and detecting the best solutions to prevent future incidents in the Regional Health Service (RHS) in Tuscany, given its proven validity and effectiveness in systems analysis of AEs [25,26], as well as ease of use for front-line clinicians [27]. It is a reactive method for learning from structured reflection on significant clinical cases, with a focus on preventable care delivery problems (CDPs) and contributory factors (CFs) [4].

The application of SEA requires the active involvement of all the actors engaged, even indirectly in case management, as well as external experts to validate the analysis. These may also provide an alternative point of view both for the study of the case and the identification of alternative and safer procedures. A facilitator trained in systems analysis of AE and communication in teams is recommended to effectively lead the process. Medical forensic analysis may be used in a complementary manner but cannot replace SEA, since it uses a jurisprudential approach that can establish a link between conduct and its consequences using a procedural logic that is often very far from the clinical truth and systems dynamics. From an administrative point of view, SEA is a confidential process, and the documents resulting from the analysis cannot be used against clinicians for disciplinary action in accordance with a regional resolution [28]. The principle of confidentiality was recently enforced by a new Italian law for patient safety, where it is recognized that “*... the minutes and the documents resulting from the management of clinical risk cannot be acquired or used as part of legal actions against healthcare professionals.*” [29].

In the case analysis performed during this study, the working groups reconstructed the timeline of the facts leading to the adverse event according to the available information, collected through press releases, individual reports, and record reviews. Then, the group evaluated the relevant healthcare delivery problems and the related factors were described in neutral language. This was later classified by a clinical expert and a specialist in ergonomics and human factors, according to the criteria of the London Protocol (Table 2) [4]. Some recommendations for prevention were also shared after the completion of the analysis and were then published in a regional guide for risk management in CM.

### 3.2. The Application of FMEA to Complementary Medicine Clinics

FMEA is a proactive risk management tool for evaluating human reliability. Basically, this tool provides a scheme that guides safety officers in the study of care delivery problems in a working process and prioritizes improvement actions in order to reduce the risk of incidents [30,31].

FMEA consists of seven steps:

(1) Selection of the significant process to be analysed;
(2) Organization of a multidisciplinary group of experts;
(3) Description of the different phases of the process;
(4) Identification of FMs for each step of the process (i.e., anything that could go wrong, including rare and minor problems);
(5) Definition of a numerical value (on a scale from 1 to 10) for frequency of the event, potential serious consequences, and probability that the healthcare providers will identify the failure. The Risk Priority Number (RPN) defined for each method of failure identified will help estimate the frequency, severity and detectability of the FMs (Table 3);
(6) For each FM, calculation of the Risk Priority Number (RPN) considering on a scale from 0 to 10 the severity (S) of the effects, the possible occurrence (O) of the cause, and the likelihood of detection (D) of the cause: PRN = O × S × D;
(7) Use of the results of PRN calculation (from 0 to 1000) to prioritise the improvement actions aimed at preventing the FMs.

The clinic “Fior di Prugna” in Florence, and the Regional Reference Centre for TCM and acupuncture in Tuscany, were selected for the application of FMEA. A group was set up consisting of acupuncture professionals representing the specialties of TCM, acupuncture techniques and nursing, after training on patient safety. The aim was to describe and analyse the risks of a complete diagnostic therapeutic and healthcare pathway of acupuncture using the FMEA approach.

## 4. Results

Below are the results of SEA in homeopathic practice (Table 4) and of the application of FMEA to the activities of the acupuncture clinic (Table 5) in order to identify the possible risks of preventable incidents.

### 4.1. SEA in Homeopathic Practice

The training programme was attended by 28 participants, out of 37 professionals employed in the regional network of CM, mostly women (68%) and medical doctors (72%).

Table 4 reports the results of the applying SEA to six cases of AE in CM that occurred in Italy between 2006 and 2009, that were presented by the participants during the training programme.

Six cases occurred amongst out-patients during their medical treatment with homeopathic or herbal products.

Three cases (1, 2, 3 in Table 4) occurred in paediatric care and resulted in patient death; two of them were related to a CDP due to medication substitution; the third resulted in a failure to prevent patient deterioration. They all involve human factors, given that they are attributable to erroneous decisions by families and providers, as well as task-related factors (lack of follow-up after the visits). In one case in particular, institutional factors were involved (professionals’ training and authorization). The suggested preventive strategies include the completion of an explicit risk–benefit analysis before the decision to prescribe CM as a first choice in fragile patients (i.e., infants) and in the case of medication substitution. Providing education and counselling to families about treatment options is also recommended.

In two cases involving adults (4 and 5 in Table 4), the consequences were a delay in the diagnosis of patient deterioration, and one case (6 in Table 4) there was an adverse reaction due to the interaction of a herbal product with ongoing cancer treatments.

In cases 1 and 5 there was definitely confusion and/or clinical error with respect to the so-called “homeopathic aggravation”, which is a temporary aggravation of the symptoms after the homeopathic prescription, followed by an improvement of general and local conditions. In this case there was confusion between a supposed positive effect of the treatment, and a progressive worsening of clinical conditions because the treatment was not effective. In case 4, the most relevant CDP concern was the timing of CM treatment initiation, which corresponded to a holiday period. The patient travelled far from home without a clear reference for support. This was due to poor planning of the care process and informed consent (organizational contributory factors), and these issues were later addressed in the recommendations for improvement. In case 5, there was a problem of inadequate diagnostic performance due to human and task-related factors, with the doctor focusing on the symptoms and not on the underlying disease. Actions for improvement include a general clinical assessment before the CM treatment and coordination of the care plan with relevant specialists when needed.

Case 6 is an example of incorrect patient behaviour due to a lack of education about the risks of dangerous interactions between conventional medicines and CM. This is attributable to institutional factors, in a context where the provision of proper information about possible interactions of herbs and access to professional consultation are not effectively covered within the healthcare system. For this reason, information for professionals and the public on the risks associated with the isolated or integrated use of CM is recommended to prevent dangerous interactions.

### 4.2. FMEA Application in the TCM Centre “Fior di Prugna”

The FMEA analysis focused on the “TCM and Acupuncture” pathway. The first step was to identify the phases of the selected route: reservation and delivery times of the visit, patient’s first meeting with healthcare professionals, first medical examination with triage, and treatment with acupuncture and TCM (“Rabbit” list) or auricular acupuncture (“Turtle” list). The analysis was then focused on the “TCM and Acupuncture” (“Rabbit” list).

For each step, the working group identified:

◦ activities and operators involved in these phases;
◦ problems and criticalities that might arise during the activities;
◦ main causes of such problems;
◦ impact of these problems on the health of the patient and on the efficacy of the service and treatment.

A value was assigned to each problem identified: occurrence (O), i.e., possibility for the problem to actually occur; severity (S), i.e., consequences of the problem; and detection (D), i.e., how easy it is for the operator or for the system to ascertain the problem.

In acupuncture, the highest RPNs are related to the long waiting list for an appoiment for the visit, the risk of infection caused by the insertion of the needles, and by the risk of failure at the recovery stage after the procedure. In this case, the solution of the problem included the use of a checklist to verify the sterilization of the needles, as well as hand hygiene before the procedure. (Table 5).

Events emerging with the highest RPN values are:

- A long waiting list due to the high number of requests, causing delayed treatment (especially for those conditions where acupuncture is the first choice). This is due mainly to high demand; the consequence was a higher risk of aggravation of the disease and unsuccessful treatment, with loss of credibility of the healthcare service.
- Error in the diagnostic approach in the “energetic diagnosis” due to lack of knowledge, or the high complexity of the disease.
- Errors in the execution of the treatment by the health professional, and patient stress or hypersensitivity, which lead to a higher risk of leaving needles in situ, errors in needle insertion, application of contraindicated methods, possible infection in the case of accidental puncture, damage from erroneous puncture, and the occurrence of biliary or renal colic.
- Finally, there is excessive relaxation of the patient after the session of acupuncture and Chinese massage, and thus there is a higher risk of accident if the patient leaves the clinic immediately, driving for instance a car without waiting for the necessary rest time. To reduce this type of risk, an adequate time of rest (at least 20 min) must be considered and a dedicated room after the acupuncture session is required.

## 5. Discussion

In CM, and particularly acupuncture and homeopathy, the possible inefficacy of the treatment (and therefore the potential risks deriving from the practice) is usually considered as a subjective error, frequently attributed to professional incompetence and insufficient training.

However, other important risk factors can be detected. In many countries, CM is provided outside the national healthcare system and is practised by non-regulated personnel. In Western countries and especially in Europe [32] accredited training courses in CM are very scarce.

Different types of self-regulation and the lack of regulatory bodies and professional organizations for some CM practices cause confusion over who should be responsible for systematically identifying CM-associated risk; moreover, in many cases, CM activities are not included in national reporting systems. Equally important is the scarcity of guidelines and “evidence-based” protocols for the different illnesses and clinical conditions for these medical therapies.

Adverse effects are underreported for various reasons, including lack of time and standardised reporting documentation, sensitivity to criticism, and unwillingness to acknowledge mistakes [33,34,35].

CM physicians cannot be experts in all of the different diseases coming to their attention, and sometimes place excessive confidence in the therapeutic methods they are using.

For all these reasons, a new robust surveillance system should be developed, combined with an investigation into how the concept of risk in CM can be established in the medical profession in order to enable full and accurate reporting of harmful incidents [36]. This is strongly felt in Italy, especially after the approval of the Law No. 24/2017 concerning the professional responsibility of medical doctors [37]. According to this law, the judge who evaluates the harm caused to the patients by a medical error should consider whether the conduct of the health professional and/or medical doctor is consistent with the guidelines specified by the corresponding scientific societies [29,37].

When CM is provided in public healthcare systems, it is especially essential to identify methods and tools to detect adverse events and risks for patients choosing these therapies.

Another key factor is communication with patients, where medical doctors should pay attention not only to the content, but also to the form of the information given. In addition, to facilitate the therapeutic relationship, there should be favourable environmental conditions in the new healthcare facilities for example, sufficient time, quietness, concentration, etc. should be provided.

A study conducted by the Tuscan Network of Integrative Medicine (now the Tuscan Centre for Integrative Medicine, included in the Clinical Governance of the Region of Tuscany) [38], focused on the relevance of doctor–patient communication, as well as on the full integration of CM in the conventional systems of reporting and education. Active surveillance has been shown to be a feasible way to explore serious adverse events associated with the use of CM in paediatrics [39]. However, the working tools indicated in this work and applied in standard care for a long time obviously cannot replace the efforts required to achieve adequate training and professional development in CM.

Moreover, the analysis of some clinical incidents shows an almost “faith-based” approach towards the healing potential of the treatment, against all common sense and in the absence of evidence in the literature. This approach has been shown in some clinical cases of “non-conventional” treatments for patients with cancer who, without receiving official anti-cancer treatments, had fatal outcomes [40] or, more recently, in the previously mentioned case of the child who died of otitis. The application of SEA in complementary medicine can help to fill the knowledge gap of doctors, patients and their families on known and unknown risks related to this practice, and on the strategies to prevent their recurrence within the health services and professional communities. It is a powerful and easy to use tool that brings a systems perspective to clinicians, provides adequate training, and is facilitated by a safety officer with knowledge of the human factors involved.

One final comment is with respect to FMEA, which in this study is applied to diagnostic–therapeutic pathways of CM. The potential risks linked to each step of these pathways should be assessed to evaluate events, consequences, severe conditions, and possible solutions aimed at reducing the risk of incidents and adverse events. This approach is fundamental to protecting patient safety and defending medical professionalism in CM. As for SEA, the application of FMEA needs specific competences to achieve a meaningful list of priorities for action. This finds its validity in the intersubjective evaluation of risks carried out by the group of peers with expert facilitation. Both tools should be part of a formal system for patient safety management to close the loop from risk identification, to systems analysis and finally, prevention [26].

## 6. Conclusions

Patient safety and clinical risk management are already of major concern among individuals using complementary medicine, and should be key issues in the process of integration of these medicines into the mainstream public health system. Significant event audit (SEA) and failure modes and effects analysis (FMEA) can be useful tools for reducing clinical risk and improving the safety of patients using acupuncture and homeopathy. More prospective studies are needed to explore the challenges and opportunities to integrate patient safety management methods into complementary medicine.

## Figures and Tables

**Table 1:** List of most common terms and concepts in pharmacovigilance (modified from the Uppsala Monitoring Centre. WHO Programme for International Drug Monitoring [24].

TERMS	CONCEPTS
Adverse drug reaction (ADR)	A harmful effect suspected to be caused by a drug. The term is properly reserved for late-stage analysis when the association between a medicine and an adverse effect has moved beyond ‘unmeasurable’ or ‘uncertain’
Adverse effect	A negative or harmful patient outcome that seems to be associated with treatment, including total ineffectiveness
Adverse event	Any negative or harmful occurrence that takes place during the process of care and that may or not be associated with a medicine.
Benefit	(a) positive therapeutic effects of treatment in an individual; (b) positive health, social or psychological effects of treatment from the patient’s perspective.
Benefit-risk or more accurately, benefit-harm	A description of positive and negative effects of a medicine and the likelihood of their occurrence, as far as they are known, as perceived by an individual.
Harm	The damage or injury that is or might be caused by a medicine, including death. The concept extends to social and psychological damage, especially from the patient’s perspective.
Hazard	The intrinsic chemical or biological characteristics of a medicine or its use that could cause harm.
Individual case safety report (ICSR)	Reports sent by health professionals or patients when an adverse effect has occurred in a patient taking one or more medicines. See also Pharmacovigilance reporting systems.
Risk	The statistical probability of harm being caused.
Serious	An adverse event or reaction that results in death; requires hospitalization or extension of hospital stay; results in persistent or significant disability or incapacity; is life-threatening.
Side-effect	Any unintended outcome that seems to be associated with treatment, including negative or positive effects.
Pharmacovigilance reporting systems	The core data-generating system of pharmacovigilance, relying on healthcare professionals and patients to identify and report any suspected adverse effects from medicines to their local or national pharmacovigilance centre or to the manufacturer.

**Table 2:** Framework for the analysis of contributory factors according to the London Protocol.

Factor Types	Contributory Inluencing Factors
Patients Factors	Condition (complexity and seriousness) Language and communication Personality and social factors
Task and Technology Factors	Task design and clarity of structure Availability and use of protocols Availability and accuracy of test results
Individual (staff) Factors	Knowledge and skills Competence Physical and mental health
Team Factors	Verbal communication Written communication Supervision and seeking help Team structure (congruence, consistency, leadership, etc.)
Environmental Work Factors	Staffing levels and skills mix Workload and shift patterns Design, availability and maintenance of equipment Administrative and managerial support Environment Physical
Organizational and Management Factors	Financial resources and constrains Organizational structure Policy, standard and goals Safety culture and priorities
Institutional Context Factors	Economic and regulatory context National health service executive Links with external organization

**Table 3:** Scale of values in failure modes and effects analysis (FMEA) to calculate the Risk Priority Number (RPN).

Scale		1	2	3	4	5	6	7	8	9	10	
Occurrence	Never occurring											Always occurring
Severity	No severity											Catastrophic
Detection	Immediately detectable											Undetectable

**Table 4:** Results of significant event audit (SEA) on six adverse events (AEs). CM: complementary medicine; CF: contributory factors.

Care Delivery Problems	Contributory Factors	Recommendations for Prevention
**Case 1—Newspaper**		
The CM treatment was initiated early given the patient’s condition (failure to prevent deterioration)	Excessive confidence in a good doctor–patient relationship (patient CF) Underestimation or confusion/clinical error with respect to homeopathic aggravation and progression of the disease, as well as possible adverse events (individual CF)	Perform a risk–benefit evaluation before the decision to prescribe CM and share a strategy to prevent the risks of deterioration with the patient or the family.
**Case 2—Newspaper**		
The doctor accepted the patients’ request to remove potential “life-saving” measures, but not well tolerated therapies (medication substitution)	Excessive trust in patients’ statements (individual CF) Lack of patient follow-up (task CF) Lack of communication with all the members of the family (individual CF)	Perform a risk–benefit evaluation before the decision to substitute an ordinary medication with CM and share with the patient AND the family a strategy to prevent deterioration
**Case 3—Newspaper**		
The non-medical professional prescribed “alternative drugs”, eliminating conventional drugs in too short a time (medication substitution)	Insufficient professional training and authorization, either for conventional or unconventional treatments (institutional CF)	Prevent referrals to non-medical professionals. Patient and family education and counselling on treatment options
**Case 4—Personal Report**		
The patient started homeopathic treatment at an inappropriate time: e.g., before the summer, during the holiday period (timing of CM treatment).	Lack of appropriate, specific, informed consent signed by the patient. (organizational CF) Limited availability, or difficulty in finding the homeopathic physician outside the working hours (organizational CF)	Consider service availability when prescribing CM and plan continuous follow-up, including patient advice to refer to a clinic in case of deterioration
**Case 5—Record Review and Personal Report**		
The doctor performed the erroneous clinical evaluation of an acute illness by telephone. The doctor accepted the patients’ request to avoid particularly invasive diagnostic tests (diagnostic performance)	Limited guarantee of the effects of homeopathy practice on a particular disease, or unawareness of the risks of certain diseases (individual CF) Excessively rigid application of the homeopathic protocol (task CF)	Perform a general clinical assessment of patient conditions before initiating a CM treatment and provide follow-up visits or referral to the relevant specialists to diagnose and treat different diseases
**Case 6—Record Review and Personal Report**		
The patient was not aware of the risks of dangerous interactions between her medications and herbal products (patient education)	Lack of a system of professional consultation and support both on the part of CM doctors and of conventional medicine specialists (institutional CF)	Provide medical doctors and patients with education and counselling on CM as isolated or integrated treatments to prevent dangerous interactions

**Table 5:** Example of FMEA application and RPN value calculation in the diagnostic–therapeutic pathway of the Acupuncture and Traditional Chinese Medicine Clinic “Fior di Prugna” (FdP) of Florence.

Activity	Actors	Failure modes	Causes	Possible Effects	O	S	D	PRN
**Booking a medical examination**								
Booking a medical examination through the Central Booking Office (CBO) (normal procedure)	Patients; CUP operators	Many problems in booking a medical examination. The CUP schedules an appointment even if the condition is not treated by the FdP * (no selection)	High demand, reduced operator availability. Poorly informed CBO operators	Delayed treatment (especially for conditions where acupuncture is the first choice). Unsuccessful treatment. Loss of credibility of the healthcare service	8	5	2	80
**Energetic diagnosis**								
Energetic diagnosis	Medical doctor and physiotherapist	Error in the diagnostic approach	Lack of knowledge, complex disease	Unsuccessful treatment, complex disease	3	5	4	60
**Treatment execution**								
Treatment execution	Medical doctor and physiotherapist	Intolerance of the patient to some methods, onset of undesirable side-effects, possibility of the health professional to get pricked or to leave needles in situ, error in needle insertion, application of a contraindicated method	Patient hypersensitivity, lack of time, health professional tiredness	Health professional and patient stress, infection in the case of accidental puncture, damage from erroneous puncture, occurrence of biliary or renal colic	5	4	3	60
**End of the treatment**								
Treatment execution	Medical doctor and physiotherapist	Excessive relaxation of the patient after the treatment.	After the treatment, the patient is immediately dismissed without waiting for a necessary rest time	A higher risk of accident if the patient leaves the clinic immediately, using a car or a bike for instance.	8	3	2	48

* FdP: Acupuncture and Traditional Chinese Medicine Outpatient Clinic, Local Health Unit Tuscany Centre, Florence.
